# Speech's syllabic rhythm and articulatory features produced under different auditory feedback conditions identify Parkinsonism

**DOI:** 10.1038/s41598-024-65974-6

**Published:** 2024-07-09

**Authors:** Ángeles Piña Méndez, Alan Taitz, Oscar Palacios Rodríguez, Ildefonso Rodríguez Leyva, M. Florencia Assaneo

**Affiliations:** 1 Faculty of Psychology, Autonomous University of San Luis Potosí, San Luis Potosí, Mexico; 2 SRI International, Menlo Park, CA USA; 3 Faculty of Medicine, Autonomous University of San Luis Potosí, San Luis Potosí, Mexico; 4 Institute of Neurobiology, National Autonomous University of Mexico, Querétaro, Mexico

**Keywords:** Peripheral neuropathies, Diagnostic markers

## Abstract

Diagnostic tests for Parkinsonism based on speech samples have shown promising results. Although abnormal auditory feedback integration during speech production and impaired rhythmic organization of speech are known in Parkinsonism, these aspects have not been incorporated into diagnostic tests. This study aimed to identify Parkinsonism using a novel speech behavioral test that involved rhythmically repeating syllables under different auditory feedback conditions. The study included 30 individuals with Parkinson's disease (PD) and 30 healthy subjects. Participants were asked to rhythmically repeat the PA-TA-KA syllable sequence, both whispering and speaking aloud under various listening conditions. The results showed that individuals with PD had difficulties in whispering and articulating under altered auditory feedback conditions, exhibited delayed speech onset, and demonstrated inconsistent rhythmic structure across trials compared to controls. These parameters were then fed into a supervised machine-learning algorithm to differentiate between the two groups. The algorithm achieved an accuracy of 85.4%, a sensitivity of 86.5%, and a specificity of 84.3%. This pilot study highlights the potential of the proposed behavioral paradigm as an objective and accessible (both in cost and time) test for identifying individuals with Parkinson's disease.

## Introduction

Parkinson's disease (PD) is a complex and frequent neurodegenerative disorder characterized by an extensive collection of motor and non-motor symptoms, a variable response to treatment, and a generally progressive course^1,2^. PD is considered a public health problem, as it is the second most common neurodegenerative disorder after Alzheimer's disease^3^. Furthermore, the Global Burden of Disease study has estimated that PD cases will double from 7 million in 2015 to 13 million in 2040^4^. Due to its complexity, variability, and subtypes, PD represents a challenge in its diagnosis based on history and physical examination^5^. Moreover, there are other tests, as imaging, to confirm the diagnosis of PD, but these tend to be expensive and unavailable in many clinical centers, especially in under-developed countries. Therefore, attention has been focused on specific, noninvasive, and low-cost biomarkers.

Dysarthria (i.e., abnormalities in different aspects of speech production) represents an early symptom of PD and atypical Parkinsonian syndromes^6,7^. In line with this observation, several studies explored the potential of different speech and voice features as PD biomarkers^8^. Several of these studies rely on the diadochokinetic task (DDK), which evaluates articulatory speech impairments by asking participants to repeat as quickly as possible a consonant–vowel combination (typically, the PA-TA-KA sequence, which involves different places of articulation: bilabial, alveolar, and velar)^9^. Based on this task, low-cost vocal tests have been developed, showing a high success rate in identifying PD in its early stages^9–11^. All these tests rely on the analysis of speech acoustic features in the millisecond scale (e.g., Mel-frequency cepstral coefficients or phonation jitter/shimmer). However, longer timescales features, such as speech articulatory rate and regularity, have also been identified as altered in PD^12^, as well as in atypical Parkinsonian syndromes^13^, but remain excluded from vocal tests. This is surprising, given that general speech rhythm features and timing organization have been found to be abnormal in PD by various studies^14–16^. Including these longer timescale features in vocal tests could provide a more comprehensive assessment of speech abnormalities in PD and it could enhance our understanding of the underlying pathophysiology of this disorder.

In addition to dysarthria, it has been shown that the effects produced on the ongoing speech by an unexpected modulation of the auditory feedback differ between individuals with PD and controls^17–19^. For example, in^17^, participants were asked to sustain vocalization of a vowel while an unexpected perturbation was introduced to the auditory feedback, changing the pitch or intensity of the produced sound. While all participants spontaneously adjusted their vocalization to compensate for the perturbation, individuals with PD exhibited larger compensatory responses than control participants. This result made researchers hypothesize that individuals with PD have abnormal speech auditory-motor integration. Furthermore, interventions based on altered auditory feedback have successfully restored speech fluency in individuals with dysarthria^20^.

Bringing together the existing literature, we developed a modified diadochokinetic task in which subjects are instructed to rhythmically repeat the syllables PA-TA-KA under different auditory feedback conditions. Using this test, we explore significant differences between individuals with Parkinson and controls in their rhythmic and articulatory speech features. Specifically, we assessed general speech features such as a subject’s ability to whisper, syllabic rhythm stability, or several syllable-level errors instead of measures of phonation, respiration or prosody as in previous studies^9,11^. Furthermore, using a supervised learning technique, we show this paradigm's high accuracy, sensibility, and specificity to identify Parkinsonism.

## Methods

### Participants

Two cohorts of gender-matched participants (individuals with PD and controls) completed this study. Both cohorts comprised Mexican subjects, all of whom were native Spanish speakers. Signed informed consent was obtained from all participants. The protocol was evaluated and approved by the Ethics Committee of the Faculty of Psychology of San Luis Potosí (Registration number: 2131082021). All methods were performed in accordance with the relevant guidelines and regulations.

A total of 28 participants (17 female and 11 male) composed the group of individuals with PD (14 rigid-akinetic and 14 with tremor). They were diagnosed by a neurologist, following the Movement Disorder Society (MDS) clinical diagnosis criteria for PD^21^. The diagnosis was made before the start of this study. The PD subtype (i.e., rigid-akinetic or tremorigenic) was identified according to the clinical manifestations (TRAP: tremor, rigidity, akinesia, and postural instability). The akinetic-rigid form was defined for participants who reported (1) no tremor at onset, (2) minimal progression of tremor since diagnosis, and 3) a negative report of tremor as a current major manifestation or impairment associated with PD. The tremorigenic form was defined for patients who presented tremor as (1) the predominant initial sign of the disease, (2) had progression in tremor severity since diagnosis, and (3) reported tremor as a current major manifestation and impairment associated with PD compared to other motor signs. Unfortunately, DAT scans were not available. The ages of the individuals with PD ranged from 38 to 88 years (mean 68.6, SD 11). We did not modify the treatment during the task; all individuals with PD were in an ON state, except for two participants who did not take their medication on the day of the study. Participants completed the Mini-Mental State Examination (MMSE)^22,23^. to assess cognitive decline. No participant scored below 21, indicating the absence of severe cognitive impairment^24^. For more clinical information about this cohort see Supplementary Table 1.

The group of controls comprised 30 participants (17 female and 13 male) who did not report having neurologic or psychiatric disorders (mean = 66, SD = 9, range 46–93).

Both cohorts of participants completed a short questionnaire about their musical experience and educational level. There were no significant differences between cohorts in the assessed demographic features (see Supplementary Fig. 1). The participants were also asked whether they had been previously diagnosed with any hearing impairments. Two control participants and two individuals with PD reported a unilateral partial hearing loss.

### Auditory stimulus

*Target audio:* The syllables “pa,” “ta,” and “ka,” spoken aloud and whispered, were recorded by a female Spanish speaker. Praat software^25^ was used to make each syllable last 250 ms. Next, five repetitions of the sequence pa-ta-ka, spoken or whispered, were concatenated, generating two rhythmic (4 syllables/sec), 3.75-s-long audio files: the whispered and the spoken targets.

*Exogenous speech:* A 5.5-s-long audio file comprising a rhythmic train of syllables was synthesized at 16 Hz using the MBROLA text-to-speech synthesizer^26^ with the Spanish Male Voice “es2.” A set of 13 Spanish syllables (“te,” “bi,” “ki,” “pu,” “bo,” “la,” “su,” “go,” “mu,” “rra,” “le,” “do,” “fe") were repeatedly and randomly concatenated to achieve the desired length. All phonemes were equal in pitch (200 Hz), and the duration was set to 0.125 ms.

*Noise:* A 5.5-s-long audio file comprising white noise was synthesized using Matlab.

### Procedure

All participants performed the experimental test in a room with low ambient noise. They sat in front of a computer wearing insert earphones (ETYMOTIC ER1), and we recorded their vocalizations with a microphone connected to the laptop (Marantz Pro M4U). All instructions appeared written on the computer screen and were verbally reinforced by the examiner.

**Figure 1:**
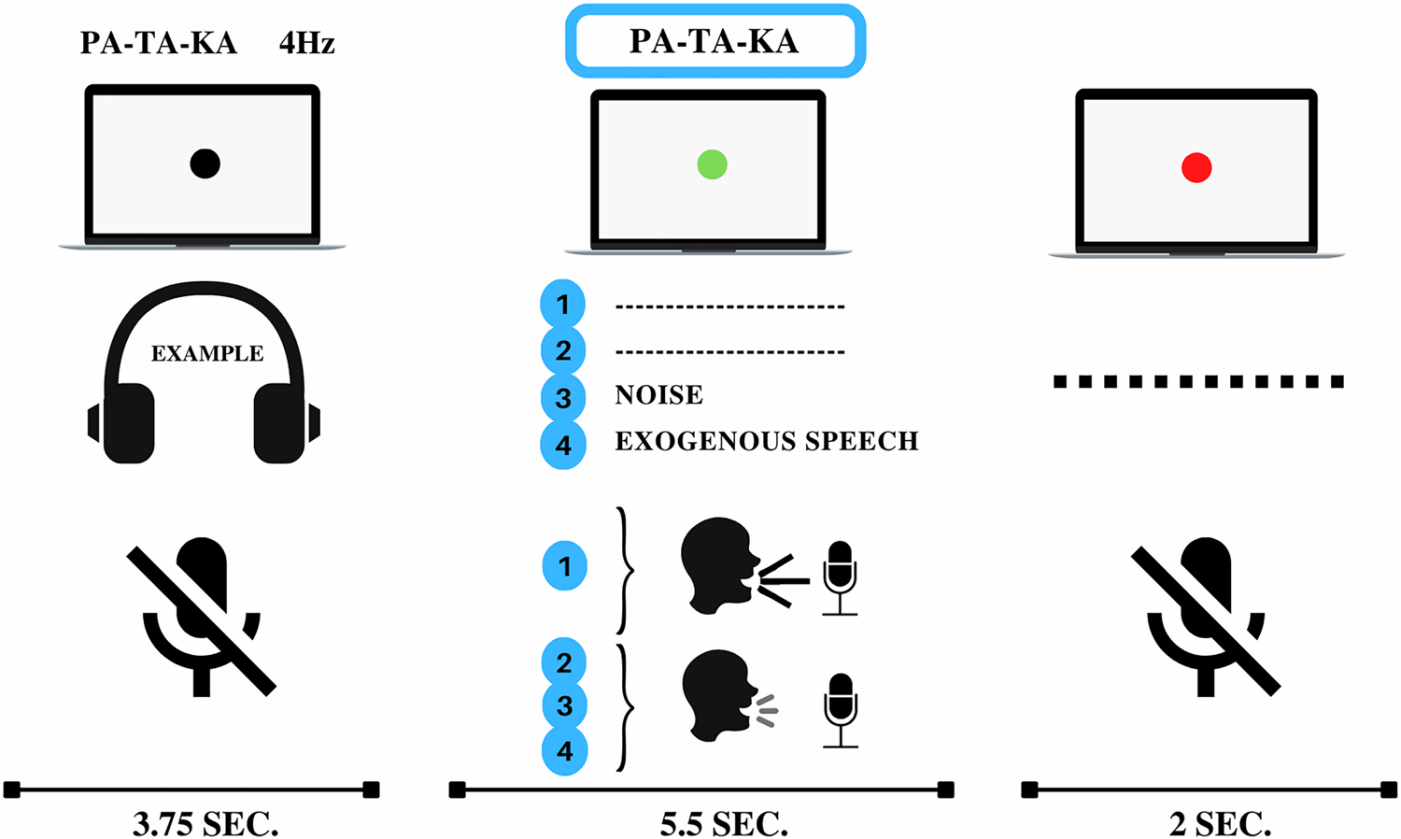
Experimental design of the test (trial structure). During the passive listening phase, participants were presented with a target audio (whispered or spoken aloud). Next, during the repetition phase, participants continuously mimicked the target audio under different auditory feedback situations. Each trial was one of four conditions according to the participant’s voice level (determined by the target audio) and the auditory feedback situation. Condition 1: Speaking aloud while listening to their voice (Normal feedback). Condition 2: Whispering while listening to their whispers (Reduced feedback). Condition 3: Whispering while listening to noise (Masked feedback). Condition 4: Whispering while listening to an alien voice speaking at the same syllabic rate (Replaced feedback). The intertrial interval was set to two seconds.

The experimental test consisted of 28 trials and had a total duration of approximately 10 min. Each trial consisted of passive listening and repetition phases (see Fig. 1). During the passive listening phase, the target audio was presented through the earplugs, and participants were instructed to pay attention to it while fixing their gaze on a black dot centered on the screen. The target audio lasted 3.75 s and comprised a repetition of the pa-ta-ka sequence, whispered or spoken aloud (see the Auditory Stimuli section). At the end of the audio playback, the dot on the screen turned green, signaling the beginning of the repetition phase. During this phase, participants were instructed to continuously echo the target audio, matching the presented rhythm and voice level (i.e., whispering or speaking aloud). After 5.5 s, the green dot turned red, prompting participants to stop vocalizing and to wait until the subsequent trial. The intertrial interval was set to two seconds.

Each trial belonged to one of four possible conditions (see Fig. 1):

1. Natural feedback: The target audio comprised loud speech, and no auditory stimulus was presented during the repetition phase.
2. Reduced feedback: The target audio comprised whispered speech, and no auditory stimulus was presented during the repetition phase.
3. Masked feedback: The target audio comprised whispered speech, and the noise audio was played during the repetition phase, masking the participant’s voice auditory feedback.
4. Replaced feedback: The target audio comprised whispered speech, and the exogenous speech audio was played during the repetition phase, masking the participant’s voice auditory feedback.

We presented seven trials per condition in a randomized order.

As stated in the introduction, previous works point to an abnormal speech auditory-motor integration in individuals with PD. Accordingly, the four conditions were designed to assess whether varying levels and types of auditory feedback affect speech features differently in individuals with Parkinson's disease compared to controls. Conditions 1, 2, and 3 (normal feedback, whisper, and noise) manipulate the amount of external auditory feedback: full, partial, and none^27^. This manipulation is based on the hypothesis that speakers use sensory feedback to control speech rate and articulatory timing^28^. Condition 4 also involves no auditory feedback but provides the subject with an external cue of the target rate. Previous research indicates that individual differences in white matter structures connecting auditory and frontal regions lead to quantitative differences in speech syllabic rhythm behaviors under this condition^29^, and the level of synchronization between produced and perceived speech has been proposed as a marker for different types of stuttering disorders^30^.

### Data preprocessing

**Figure 2:**
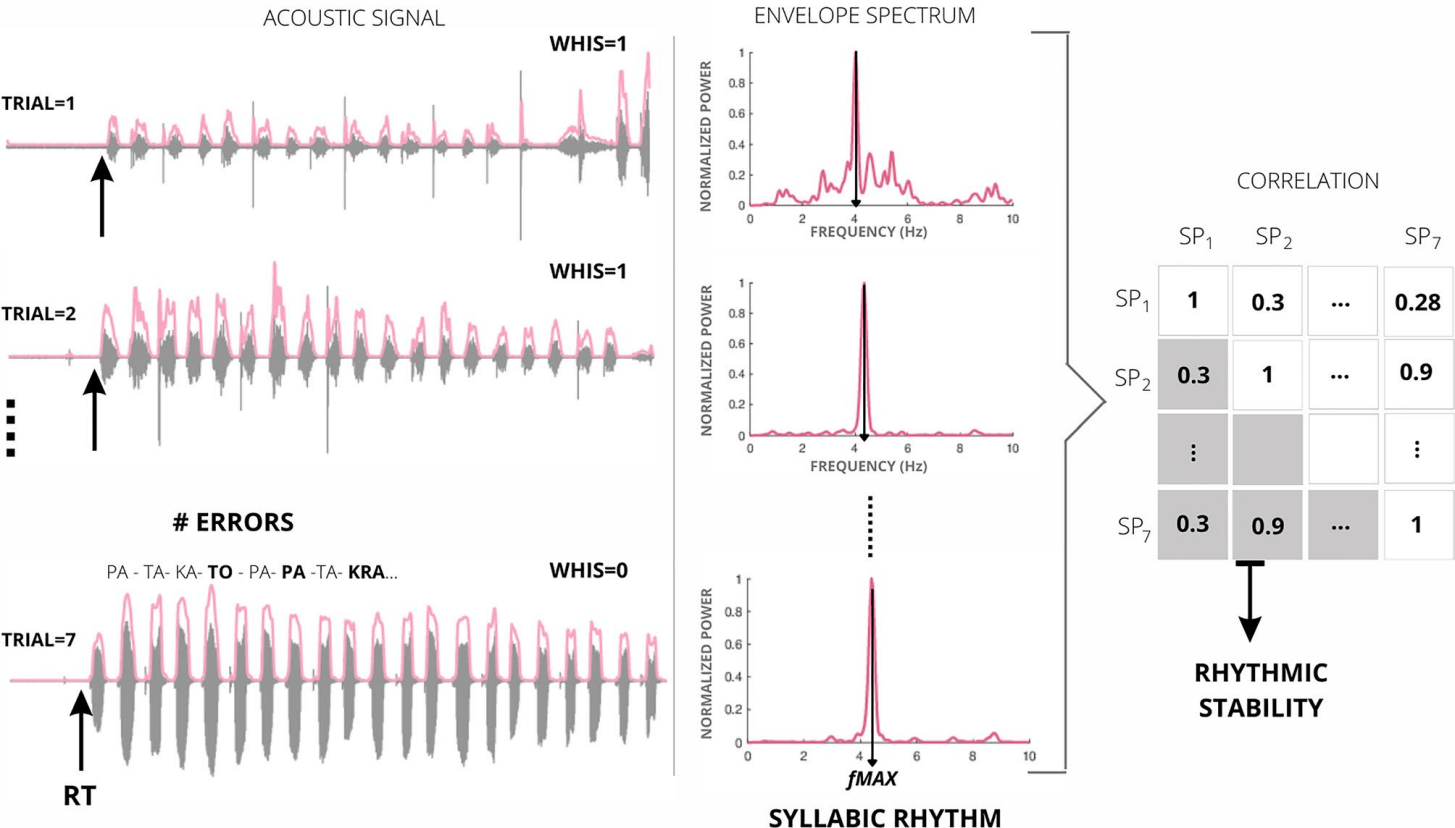
Data preprocessing: a sketch of the pipeline applied to one condition for one representative participant. The left column shows the acoustic signal of each trial in gray, with its corresponding envelope over-imposed in pink. Trials 1 and 2 are whispered (Whis = 1), and trial seven is voiced (Whis = 0). Arrows point to the reaction time. The middle column displays the spectrum of the envelope for each test. Arrows indicate the syllabic rate (fMAX). The right panel shows the correlation matrix of the envelope spectra (Sp_1–7_) with its lower diagonal elements highlighted in gray. The rhythmic structure consistency across trials was computed as the average of these elements.

For each participant and trial condition, five parameters were extracted (see Fig. 2): (1) the number of whispered trials, (2) the number of speech errors, (3) the mean reaction time, (4) the mean syllabic rate, and (5) the rhythmic structure consistency across trials.

The first author manually computed the number of whispered trials and speech errors. Praat software^25^ was used to listen to and visualize the acoustic signals. Each trial was categorized as whispered if voiced speech (i.e., with activation of the vocal folds) occurred for less than 0.55 s (10% of the trial length). An error was identified if the participant: (1) repeated a syllable (e.g., “pa-ta-**ta**-ka”), (2) exchanged a syllable (e.g., “pa-**ka-ta**”), or if a syllable was wrongly articulated (e.g., “pa-**tra**-ka”).

Speech onset time was automatically extracted from each trial using Praat. Using the “Annotate: To Text Grid (silences)” command each time point has been identify as containing silence or speech; the first speech point preceded by a silence has been identify as the speech onset. Reaction time was computed as the average of the speech onset across trials of the same condition.

To estimate the rhythmic features of the spoken samples, we calculated the speech envelopes as the absolute value of the acoustic signal’s Hilbert transform^25^. Subsequently, we used the fast Fourier transform to extract the spectrum of each trial’s envelope. Each trial's syllabic rate was estimated as the frequency value with the maximal power (see Fig. 2). The estimated syllabic rates were averaged across tests of the same condition.

It’s important to mention that the speech envelope has been widely been explore in the cognitive neuroscience field giving its role played during speech comprehension^31,32^, as well as a medium to explore the speech rhythm stability across languages^33,34^. Furthermore, since the speech samples obtained with this protocol contain a repetition of a sequence of non-coarticulated syllables, each cycle in the speech envelope represents one syllable (as shown in the left panels of Fig. 2). Accordingly, the syllabic rate computed as the frequency with the maximum power in the spectrum of the speech envelope closely matches the articulatory rate, a metric previously used by other authors to explore speech rhythm.

Next, to explore the stability of the rhythmic structure of the envelope across trials of the same condition, we computed the correlation matrix between their envelope spectra. The rhythmic structure consistency across trials was estimated as the mean value of the lower diagonal elements of the matrix (see Fig. 2).

### Data analysis

*Statistical comparisons:* Non-parametric inferential statistics compared the individuals with PD group with the controls. More specifically, we used the Mann–Whitney test for two independent samples. All reported p-values were corrected using a false discovery rate approach^26^ for multiple comparisons. Effect sizes were determined using rank-biserial correlations, which can be interpreted as the disparity between the proportion of favorable and unfavorable evidence^35^. In our study, all effect sizes exceed r = 0.37. For instance, an r value of 0.38 indicates that favorable evidence outweighs unfavorable evidence by a ratio of 69% to 31%. All procedures were carried out in Matlab.

*Machine learning:* A random forest classifier model was trained on the binary classes of individuals with PD and controls using the data obtained from the five evaluated parameters. The metrics sensitivity, specificity, and accuracy were considered to assess the model’s performance. The leave-one-out cross-validation method was used to assess the model generalization performance. This method consists of several iterations of the training–testing data sets, where in each iteration, one participant is selected to test the model, and all others are used to train it. All plausible training–testing combinations were evaluated, and the predicted outcomes were used to compute the accuracy, sensitivity, and specificity. This process was repeated 100 times, and the results were averaged. This process was conducted using the *sklearn* library in Python^36^.

## Results

We assessed the goodness of a behavioral test based on speech samples to differentiate individuals with PD from controls. Thus, we evaluated individuals with PD and controls in a task consisting of continuously and rhythmically (four syllables per second) repeating the syllables “pa-ta-ka” under different auditory feedback conditions: Normal (speaking aloud while hearing the produced sounds), Reduced (whispering while hearing the produced sounds), Masked (whispering while hearing white noise), Replaced (whispering while hearing an alien voice). While the normal condition represents an adapted version of the DDK task used in previous studies^9^ (the task was modified by presenting to the participants a target syllabic rhythm), the other three integrate a modulation in the participants’ voice feedback. For the Reduced condition, feedback is not entirely removed but diminished, given the whisper-low volume. In the Masked and Replaced conditions, the feedback is completely blocked, but in the last one, participants get an external cue of the intended syllabic rate. It has been shown in healthy participants that whispering while listening to an external stable rhythm leads some individuals to synchronize the produced syllabic pace^29^. Additionally, individuals with PD show abnormal rhythmic speech entrainment with a model speaker^37^.

From the obtained recordings, we computed five parameters for each feedback condition: (1) whispering ability, (2) the number of articulatory errors, (3) reaction time to initiate speech, (4) syllabic rhythm, and (5) rhythmic structure consistency across trials (for more details, see the Methods section). Two different analyses were conducted on these parameters. First, we compared the parameters for individuals with PD and controls across feedback conditions. This allowed us to identify the speech features being abnormal in individuals with PD. Secondly, we fed the parameters to a supervised learning algorithm to assess the predictive power of the parameters’ combination to differentiate individuals with PD from control participants.

**Figure 3:**
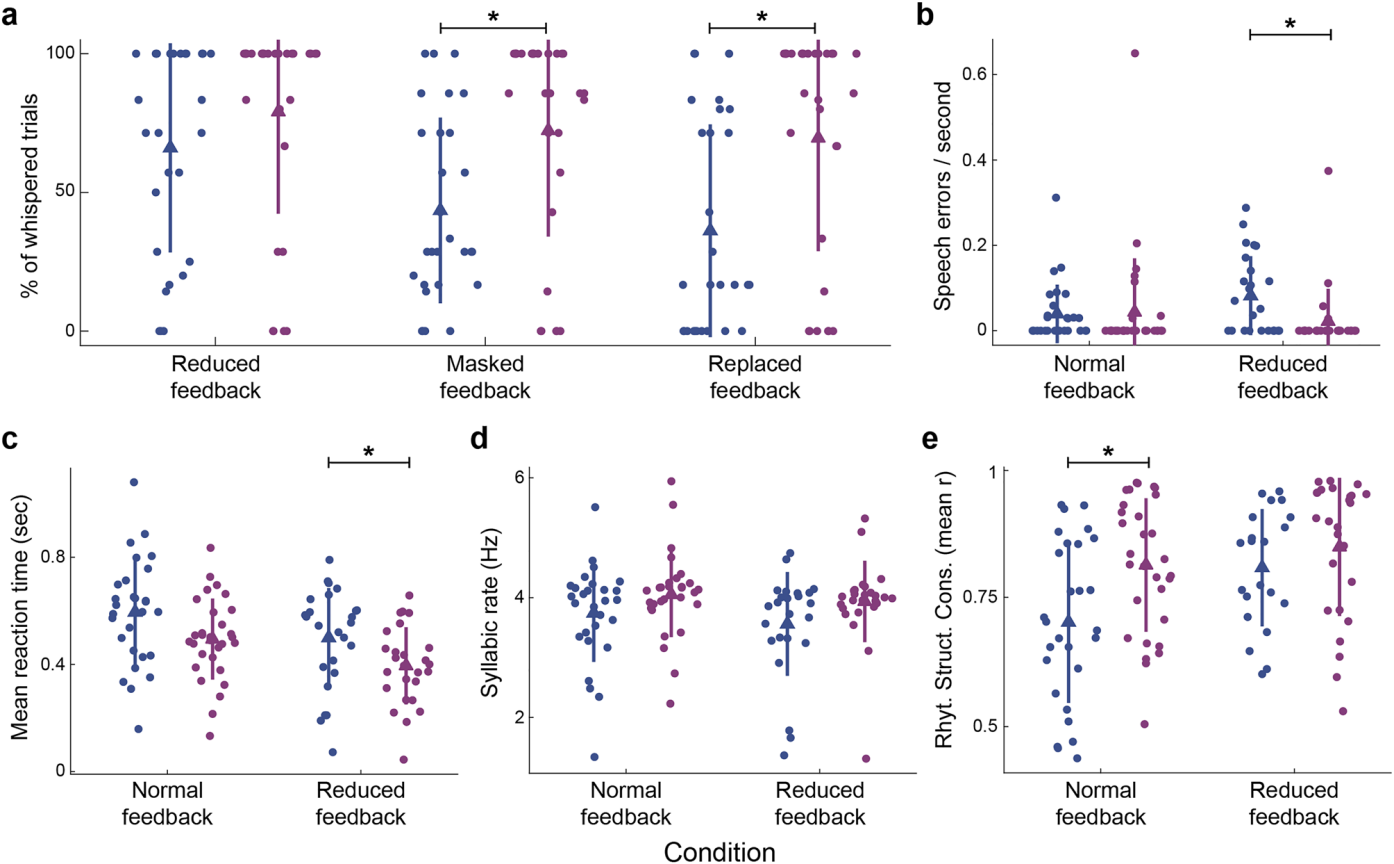
Statistical comparisons between the extracted parameters of individuals with PD and healthy controls. (**a**) Percentage of whispered trials across conditions where whispering was required. (**b**–**e**) Errors per second, mean reaction time, mean syllabic rate, and rhythmic structure consistency across trials, respectively, across Normal and Reduced feedback conditions. In all panels, colors identify the different groups; dots represent individual subjects (blue: Ind. with PD, purple: controls), bars represent the standard deviation, triangles represent the mean values, and *p < 0.05.

To start, we explored if individuals with PD could correctly follow the instructions or if they showed more difficulties than control participants. Specifically, we investigated whether they could whisper in the conditions with this requirement (i.e., Reduced, Masked, and Replaced feedback conditions). We compared the percentage of whispered trials between individuals with PD and controls. The results showed significant differences between the group of individuals with PD and control subjects in the Masked and Replaced feedback conditions (see Fig. 3a; Reduced: Ind. with PD: M = 66%, SD = 38%; Control: M = 79%, SD = 37%; *p* = 0.102; Masked: Ind. with PD: M = 43%, SD = 33%; Control: M = 72%, SD = 38%; *p* = 0.007 and r = 0.43, Rank-Biserial Correlation; Replaced: Ind. with PD: M = 36%, SD = 38%; Control: M = 70%, SD = 41%; *p* = 0.007 and r = 0.44, Rank-Biserial Correlation). Individuals with PD had difficulties in whispering the syllables when presented with altered feedback. Given that the number of whispered trials in the Masked and Replaced feedback conditions significantly differed between individuals with PD and controls, differences in the rest of the parameters were only assessed between Normal and Reduced feedback conditions. This was done because groups differ in the number of spoken-aloud trials for the other feedback conditions, making it impossible to disentangle whether the differences (if observed) derive from the auditory feedback state situation or from speaking aloud.

For all other explored parameters, we found that: (i) individuals with PD made more speech errors than controls, only for the Reduced feedback condition (see Fig. 3b; Normal: Ind. with PD: M = 0.039 err/sec, SD = 0.069 err/sec; Control: M = 0.044 err/sec, SD = 0.126 err/sec; *p* = 0.142; Reduced: Ind. with PD: M = 0.082 err/sec, SD = 0.092 err/sec; Control: M = 0.022 err/sec, SD = 0.076 err/sec; *p* = 0.004 and r = -0.43, Rank-Biserial Correlation); (ii) individuals with PD had slower reaction times than controls, only for the Reduced feedback condition (see Fig. 3c; Normal: Ind. with PD: M = 0.59 s, SD = 0.20 s; Control: M = 0.49 s, SD = 0.15 s; *p* = 0.088; Reduced: Ind. with PD: M = 0.50 s, SD = 0.18 s; Control: M = 0.39 s, SD = 0.14 s; *p* = 0.044 and r = -0.38, Rank-Biserial Correlation); (iii) there was no significant difference between groups in their mean syllabic rate (see Fig. 3d; Normal: Ind. with PD: M = 3.74 Hz, SD = 0.81 Hz; Control: M = 4.05 Hz, SD = 0.71 Hz; *p* = 0.175; Reduced: Ind. with PD: M = 3.56 Hz, SD = 0.87 Hz; Control: M = 3.94 Hz, SD = 0.68 Hz; *p* = 0.175); and (iv) individuals with PD showed less rhythmic structure consistency across trials than controls for the Normal feedback condition (see Fig. 3e; Normal: Ind. with PD: M = 0.70, SD = 0.16; Control: M = 0.82, SD = 0.13; *p* = 0.030 and r = 0.41, Rank-Biserial Correlation; Reduced: Ind. with PD: M = 0.81, SD = 0.11; Control: M = 0.85, SD = 0.13; *p* = 0.128).

Once the differences between individuals with PD and controls had been established by statistically comparing the parameters’ distributions, we fed the relevant parameters into a random forest classifier to differentiate between groups (see Methods). This procedure allowed us to estimate the predictive power of these speech features to identify individuals with PD from the general population and generalize the reported results. The five variables showing significant differences between groups are the percentage of whispering trials in Masked feedback, rate of whispering tests in Replaced feedback, speech error per second in Reduced feedback, mean reaction time in Reduced feedback, and rhythmic stability in Normal feedback. First, we trained and tested the classifier with all five variables and computed accuracy, specificity, and sensitivity (see Table 1, Model 1). Given that the two altered feedback conditions only contributed to the percentage of whispered trials, we explored if both were increasing the predictive power of the instrument or if they carried redundant information. To do so, we evaluated the classifier with the percentage of whispered trials in only one or the other condition (see Table 1, Models 2 and 3). Results show that performance does not increase by including both conditions, indicating that the Masked feedback condition can be removed from the test (see Table 1, where Model 2 that includes Replaced and removes Masked Feedback performs better than Model 3 that keeps Masked and removes Replaced). Finally, we tested whether the participants’ age helped the classifier's performance, which was not the case (see Table 1, Model 4).

**Table 1:** Performance of the random forest classifier trained and tested on different sets of parameters.

Model	Parameters fed into the classifier	Accuracy	Sensitivity	Specificity
1	%whis,RepF + %whis,MF + Sp.Err, RedF + RT, RedF + Rhy.Str.Cons, NF	82.1	81.1	83
2	%whis,RepF + Sp.Err, RedF + RT, RedF + Rhy.Str.Cons, NF	85.4	86.5	84.3
3	%whis,MF + Sp.Err, RedF + RT, RedF + Rhy.Str.Cons, NF	83.3	83	83.5
4	%whis,RepF + Sp.Err, RedF + RT, RedF + Rhy.Str.Cons,NF + age	81	77	83.1

Parameters are specified as pairs of *features and condition*s. Features are as follows: whis, percentage of whispered trials; Sp. Err, speech errors per second; RT, mean reaction time; Rhy. Str. Cons., rhythmic structure consistency across trials. Conditions are as follows: NF, Normal feedback; Red. F, Reduced feedback; MF, Masked feedback; Rep.F, Replaced feedback. Accuracy, sensitivity, and specificity are reported as a percentage.

## Discussion

The present research focused on identifying speech impairments in participants with PD under different auditory feedback conditions. More precisely, five parameters were studied: 1) whispering ability, 2) the number of articulatory errors, 3) reaction time to initiate speech, 4) syllabic rhythm, and 5) rhythmic structure consistency across trials. This is proposed as a pilot phase for developing an objective diagnostic test.

The participant's ability to whisper in Reduced, Masked, and Replaced feedback conditions was analyzed, and it was found that the patient group presented more difficulties than the control group in adapting their speech output (i.e., whispering) when their auditory feedback was modified. Given that whispering was not entirely impaired in the patient group and that the difference between groups appeared only when the auditory feedback was masked or replaced, it can be inferred that the individuals with PD have an abnormal enhancement of the Lombard effect (i.e., an increase in voice intensity in response to an increase in the ambient noise level). It has been suggested that (i) the Lombard effect occurs unconsciously, driven by a subcortical mechanism, and (ii) the activation of such a subcortical mechanism can be modulated by a cortical network, allowing voluntary control of the effect^38^. The pattern of results obtained in this study suggests that the cortical network is affected in the Parkinsonian cohort: when instructed to whisper, controls, but not individuals with PD, can suppress the Lombard effect and manage to maintain low speech intensity in noisy environments (i.e., listening to white noise in the Masked feedback condition and an alien voice in the Replaced feedback condition). In line with this hypothesis, it has been shown that individuals with PD have decreased activity in frontotemporal regions^39^, and studies based on deep brain stimulation in individuals with PD provide evidence that the connectivity between frontal and sub-thalamic brain regions explains individual differences in inhibition^40^.

Articulatory errors are prevalent in the patient group when whispering the syllables. It has been suggested that individuals with PD rely more on auditory feedback due to impaired input motor control and somatosensory feedback^41,42^, which can explain the present findings. As there is no vocal fold vibration during whispering, it is more difficult for the acoustic signal to be perceived by the auditory system^43^, and speech-motor production relies on the forward model and somatosensory feedback, mechanisms proposed to be abnormal in individuals with PD. Accordingly, studies based on repetitive transcranial magnetic stimulation have shown that stimulating the auditory cortex improves articulation in individuals with PD during an overt-speech task^42^.

It was identified that the individuals with PD recruited in our study have difficulty initiating the motor process, such as articulating syllables. Furthermore, the increase in reaction time when starting speech occurs regardless of the condition in which they perform the task (Normal and Reduced feedback). The findings are consistent with the literature; different referents mention that individuals with PD struggle to initiate movement^40,44,45^.

Regarding rhythmic features, our study found that while syllabic rhythm did not show significant differences between groups across any feedback condition, there was a significant decrease in the rhythmic structure consistency (i.e., how similar the envelope’s spectrum was across trials) among individuals with PD. The existing literature on in individuals with PD speech rhythm is marked by contradictory findings. Some studies report a slowdown in speech rhythm^46^, while others report an increase^47^, and still others find no significant difference between individuals with PD and controls^48^. Our research contributes to this body of knowledge by showing that, while absolute rhythm did not differentiate between individuals with PD and controls, rhythmic structure consistency did. This suggests that rhythmic structure consistency may be a more precise measure for differentiating individuals with PD from controls, than absolute syllabic rhythm.

Contrary to the pattern of results obtained for the articulatory errors, rhythmic stability significantly differed between groups for the Normal feedback but not the Reduced feedback condition. This contrast suggests that the circuit responsible for monitoring and correcting articulatory errors does not overlap with the one supporting the stability of the speech rhythm. It has been proposed that speech rhythm emerges due to the biophysical properties of the brain areas in charge of generating speech^49^, and the interaction of motor and auditory areas^50^ modulates it. Under this framework, the observation that the rhythm becomes unstable when increasing the auditory feedback hints towards an abnormal cortical interaction between the frontal and temporal regions.

Additionally, the distribution of the disease duration across our participants is skewed to the left (see Supplementary Table 1 where 9 out of 29 participants have been diagnosed one year ago). This suggests that the abnormal speech pattern reported here are already altered at early disease stages when individuals are slightly affected by motor impairments.

The main goal of the current study was to assess the goodness of a short and easy-to-implement behavioral screening test based on speech samples. Similar designs to identify PD^11,51,52^, and atypical parkinsonism^13^, have been previously reported in the literature with promising results. In those studies, researchers typically asked participants to complete different speech tasks, such as sustained phonation, running speech, and, as in the current work, the DDK task (i.e., continuously repeating the syllables /pa/ /ta/ /ka/). They also extracted different acoustic parameters from the speech samples and entered them into a machine-learning algorithm to distinguish individuals with PD from healthy controls. Such strategies obtained high accuracy values from 85 to 99%. Two main aspects differentiate the current work from previous studies. On the one hand, although it has been shown that modified auditory feedback impacts individuals with PD differently from controls^17–19^, this observation has not been previously included in the design of speech-based screening tests. We integrate this observation by asking participants to complete an adapted version of the DDK task under different auditory feedback conditions. On the other hand, we evaluated speech features on a different time scale than those typically computed (e.g., formant periodicity correlations, Mel-frequency cepstral coefficients, phonation jitter, phonation shimmer phonation noise, and voice fundamental frequency variations^52^). Here, we focused on general speech features, such as whispering, throughout the trial or syllabic scale characteristics, such as rhythm stability or number of errors at the syllabic level. A machine learning algorithm trained on such parameters to differentiate Parkinsonian individuals with PD from controls shows accuracy within the range of previously reported results^53^. These results present novel experimental conditions (e.g., whispering, speaking under different listening conditions, and trained syllabic rate) and parameters (e.g., whispered trials, reaction time, rhythm stability, and speech errors at the syllabic level) as valuable tools to be introduced into existing screening behavioral tests to identify Parkinsonism. Additionally, it is important to highlight the accessibility of the piloted test, which only requires a standard PC, a set of headphones, and a microphone and lasts less than 4 min (i.e., only three of the four evaluated conditions are required, and each condition comprises seven 11.25-s trials). Furthermore, results can be easily extracted from the registered audio files. While RT and Rhythm Structure Consistency across trials can be automatically computed, percentage of Whispered Trials and Speech errors just required the experimenter to listen to fourteen 5.5 s long audio files (7 corresponding to the Replaced Feedback condition, to extract the percentage of Whispered Trials; and 7 of the Reduced Feedback to compute Speech errors).

A limitation of the present study is its small sample size (28 individuals with PD and 30 controls). However, this sample size is sufficient for a pilot study and reaches statistical significance. Furthermore, despite the small sample size, the current work addresses the need for cross-linguistic studies of dysarthria in PD^54^ (i.e., this study was conducted on Mexican participants, a Spanish-speaking population typically overlooked in the existing literature). Another potential limitation is the lack of control over the influence of levodopa or other medications since the individuals with PD were on medication during the application of the instrument. There needs to be more interest in addressing this issue, which makes it difficult to access reliable data and collaborate with other institutions. However, some studies suggest that speech fluency (or the lack of it) in individuals with PD is not modulated by levodopa^55–57^.

The present protocol studies rhythmic and articulatory changes related to PD in the speech production system. Although it does not address any therapeutic strategy or alternative treatment, it lays the foundation for developing noninvasive, low-cost, and easy-to-apply diagnostic tests.

## Conclusions

Syllabic rhythm stability, reaction time, whispering ability, and syllable-level articulatory errors under different auditory feedback conditions differentiate individuals with PD from controls. An automatic detection algorithm trained on these parameters showed an accuracy of 85.4% in distinguishing individuals with PD from controls. The current work represents a pilot trial, showing the potential of the introduced behavioral paradigm as an objective and accessible (in cost and time) test to differentiate individuals with Parkinson from the general population.

### Supplementary Information

Supplementary Information.

## Data Availability

The data supporting the findings of this study are available as Supplementary Data. All other data and computer code used to generate results are available upon request from the corresponding author.
