# Supplementary material for: Speech's syllabic rhythm and articulatory features produced under different auditory feedback conditions identify Parkinsonism
Source: Sci Rep. 2024 Jul 9;14:15787. doi: 10.1038/s41598-024-65974-6 (PMC11233651; doi:10.1038/s41598-024-65974-6)
Supplement: Supplementary file 1 — Supplementary Information. [file 41598_2024_65974_MOESM1_ESM.docx]

**Speech's syllabic rhythm and articulatory features produced under different auditory feedback conditions identify Parkinsonism.**

Ángeles Piña Méndez^1^, Alan Taitz^2^, Oscar Palacios Rodríguez^1^, Ildefonso Rodríguez Leyva^3^ & M. Florencia Assaneo^4*^

^1^ Faculty of Psychology, Autonomous University of San Luis Potosí, San Luis Potosí, México

^2^ SRI International, Menlo Park, CA, US (This work was performed working at Everything ALS)

^3^ Faculty of Medicine, Autonomous University of San Luis Potosí, San Luis Potosí, México

^4^ Institute of Neurobiology, National Autonomous University of Mexico, Querétaro, México.

* Corresponding author ([fassaneo@inb.unam.mx](mailto:fassaneo@inb.unam.mx))


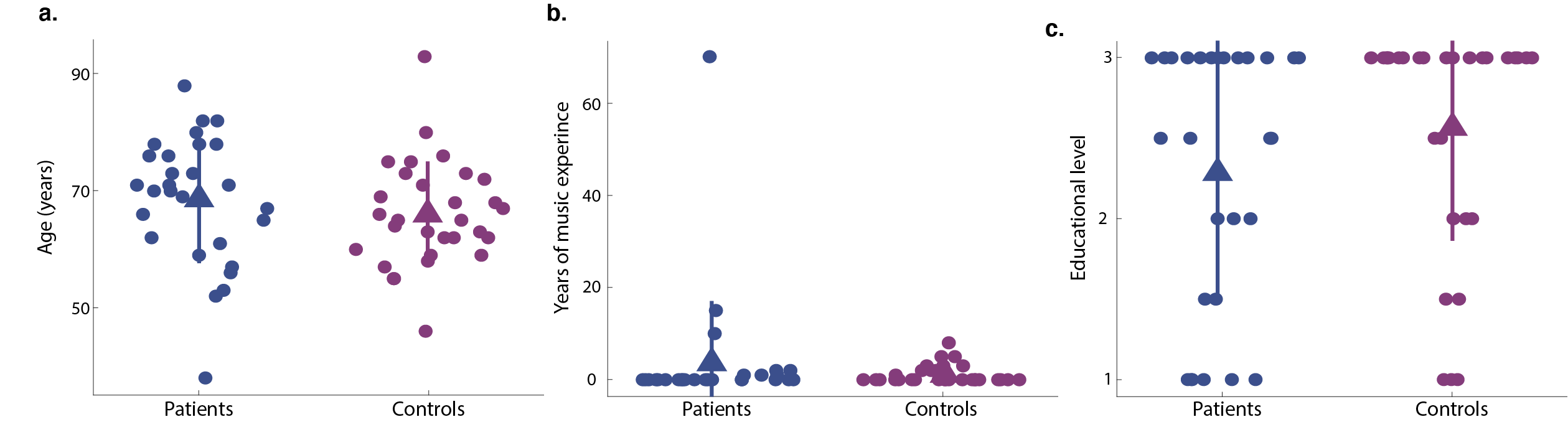


**Supplementary Figure 1; Comparison between patients and controls demographic features. a.** No significant difference was found in age distribution (Patients: M=68.3 years, SD=10.8; Controls: M=65.4 years, SD=8.9; p=0.12). **b.** No significant difference was found in years of music experience (Patients: M=3.8 years, SD=13.1; Controls: M=1.1 years, SD=2; p=0.82). **c.** No significant difference was found in educational level (Patients: M=2.3 years, SD=0.8; Controls: M=2.6, SD=0.7; p=0.22). 1: Completed elementary school. 1.5: Started but not finished high school. 2: Completed high school. 2.5: Started but not finished bachelor’s degree. 3: Completed bachelor’s degree. In all panels, dots represent individual subjects (blue: individuals with PD, purple: controls), bars represent the standard deviation and triangles represent the mean values.

| **ID** | **Type** | **Years** | **H&Y** |
| --- | --- | --- | --- |
| pp02 | rigid | 2 | 1 |
| pp03 | tremor | 17 | 5 |
| pp04 | tremor | 6 | 1 |
| pp06 | rigid | 1 | 1 |
| pp07 | tremor | 4 | 1 |
| pp08 | tremor | 1 | 1 |
| pp09 | rigid | 6 | 1 |
| pp10 | rigid | 3 | 1 |
| pp11 | rigid | 4 | 1 |
| pp12 | rigid | 2 | 1 |
| pp13 | tremor | 2 | 1 |
| pp14 | rigid | 3 | 1 |
| pp15 | rigid | 1 | 1 |
| pp16 | tremor | 1 | 1 |
| pp17 | tremor | 1 | 1 |
| pp18 | tremor | 9 | 3 |
| pp19 | rigid | 1 | 1 |
| pp20 | rigid | 4 | 1 |
| pp21 | tremor | 2 | 1 |
| pp22 | rigid | 1 | 1 |
| pp23 | tremor | 4 | 1 |
| pp24 | tremor | 1 | 1 |
| pp25 | rigid | 1 | 1 |
| pp28 | tremor | 5 | 1 |
| pp29 | tremor | 3 | 1 |
| pp30 | rigid | 12 | 4 |
| pp31 | tremor | 6 | 1 |
| pp34 | rigid | 6 | 1 |

**Supplementary Table 1; Clinical information of the cohort of participants with PD.** ID: participant’s ID; type: predominant symptom rigid-akinetic or tremor; years: years since diagnosis; motor: severity of the motor impairment; speech: severity of the speech impairment. H&Y: the Hoenh and Yahr (HY) scale stage, a scale used to determine the severity of PD^1^. It has been shown that this scale correlates with motor performance degradation^2,3^ and speech and swallowing impairment, typically arising starting from stage 2^4^.

**References**

1. Goetz, C. G. *et al.* Movement Disorder Society Task Force report on the Hoehn and Yahr staging scale: Status and recommendations The Movement Disorder Society Task Force on rating scales for Parkinson’s disease. *Movement Disorders* **19**, 1020–1028 (2004).

2. Skorvanek, M. *et al.* Differences in MDS-UPDRS Scores Based on Hoehn and Yahr Stage and Disease Duration. *Movement Disorders Clinical Practice* **4**, 536–544 (2017).

3. Reynolds, N. C., Jr & Montgomery, G. K. Factor Analysis of Parkinson’s Impairment: An Evaluation of the Final Common Pathway. *Archives of Neurology* **44**, 1013–1016 (1987).

4. Skodda, S., Grönheit, W., Mancinelli, N. & Schlegel, U. Progression of Voice and Speech Impairment in the Course of Parkinson’s Disease: A Longitudinal Study. *Parkinson’s Disease* **2013**, e389195 (2013).
